# Setting Priorities in Child Health Research Investments for South Africa

**DOI:** 10.1371/journal.pmed.0040259

**Published:** 2007-08-28

**Authors:** Mark Tomlinson, Mickey Chopra, David Sanders, Debbie Bradshaw, Michael Hendricks, David Greenfield, Robert E Black, Shams El Arifeen, Igor Rudan

## Abstract

Nearly 100,000 children under 5 years die annually in South Africa. This paper defines health research priorities to address this unacceptably high mortality rate.

Research can play a critical role in the response to global health challenges. But when needed to assist decisions on defining the priorities for health research investments. An early attempt at the global level to define health research priorities was made through the Commission on Health Research for Development in 1990 [1]. A number of subsequent initiatives addressed this problem by attempting to set priorities in global health research [2–4]. However, these approaches promoted funding for research predominantly focused upon the generation of new technologies, knowledge, and processes. Research concerned with implementation of already proven technologies and interventions was downplayed [5].

The persisting unacceptable burden of under-age-5 mortality of 10.6 million each year [6], which could be reduced by up to 63% with existing low-cost interventions [7], points to the potential role health research could play. The lack of systematic prioritization of child research funding perpetuates the neglect of interest in research on how to implement cost-effective interventions. Delivery of interventions is rarely considered a research priority; research on creating new interventions far exceeds that on delivering existing ones [5]. The Child Health and Nutrition Research Initiative (CHNRI) has developed a systematic methodology for setting priorities in health research investments that can be applied at global and national levels and for different purposes (addressing a disease, group of diseases, risk factors, etc.) [5]. The proposed major conceptual advance of CHNRI's methodology is the recognition that there should be a broader definition of health research as an activity that is not limited to generating new knowledge, but also has a vision of implementation that should help to reduce present disease burden. The methodology also attempts to systematically incorporate wider societal values and priorities.

**Summary Points**

- This paper aims to define health research priorities in South Africa, where it is estimated that nearly 100,000 children under 5 years of age still die each year.
- The authors applied the methodology for setting priorities in health research investments recently developed by Child Health and Nutrition Research Initiative (CHNRI).
- The predominant research priorities identified within the existing South African context were health policy and systems research activities to generate new knowledge on improving delivery of the simplest and most cost-effective existing interventions.
- Vitamin A supplementation was ranked first, followed by hand washing, antibiotics for pneumonia, prevention of mother-to-child HIV transmission (PMTCT), and exclusive breast-feeding.
- The CHNRI methodology has the power to discriminate among many competing research options using a simple conceptual framework.

This article reports on the first experience with country-level implementation of this new methodology in South Africa. It is estimated that nearly 100,000 children under 5 years of age still die each year in South Africa. The overall infant mortality rate of 59 per 1,000 live births has been rising over the last decade [8]. This rate also masks great variation within the country with some districts reporting an infant mortality rate of 28 and others 68 per 1,000 live births [9]. HIV/AIDS is now the leading cause of under-age-5 deaths (40.3%). Diarrhoeal disease, lower respiratory tract infections, and malnutrition, when adjusted for HIV/AIDS comorbidity, are together responsible for 20.3% of all under-age-5 deaths [10]. The two aims of the study were: (1) to determine whether the CHNRI methodology was implementable for setting child-health care priorities at the national level, and (2) to identify “child health priorities” in South Africa.

## Methods

**Activities of the technical working group.** The rationale for CHNRI methodology and its conceptual framework, application guidelines, and strategies to address the needs of the stakeholders have all been described in detail elsewhere [5,11]. Box 1 presents the elements of the methodology at a glance. In the first step, a group of six leading South African technical experts in the area of child health formed a technical working group (TWG). All are involved in national policy development and have experience working in both rural and urban settings across South Africa. Four of the experts are child health specialists with three currently engaged mostly in clinical practice, one is a clinical child psychologist, and the sixth is a medical demographer. Only two of the six have qualifications or expertise in public health. Because of valid concerns that the choice of TWG members may significantly affect the outcomes of the process, we discuss how this issue is addressed in the CHNRI methodology under the “limitations” section below.

Context was defined by the TWG in space as national (South Africa), in time as the next 10 years, target population as children below 5 years of age, and target disease burden as all cases of child deaths expected to occur within that period in those under age 5. The seven leading causes of death that account for more than 90% of child deaths in South Africa were then identified: HIV/AIDS, pneumonia, diarrhoea, neonatal causes, malnutrition, accidents and injuries, and congenital and genetic disorders. The health research domains in which research options were listed were: health policy and systems research (HPSR) to improve efficiency of current health care systems; research on improvements to existing interventions in terms of affordability and deliverability, and development of new interventions. Experts on each of the seven causes of death within South Africa were asked to select, for each of these health research domains, three research options that would, in their opinion, stand the best chance of being considered a research investment priority when evaluated against the research options addressing other causes of death. These experts had the opportunity to promote the same number of research options regardless of their relative share in disease burden, which made research options related to minor components of disease burden more likely to be over-represented rather than under-represented on the list.

The next step was the scoring of all research options by the six technical experts independently. Each of the 63 research options were assessed with regard to five criteria: likelihood that the question can be answered using ethical methods; efficacy and effectiveness; deliverability, affordability, and sustainability; maximum potential to reduce the existing child mortality burden; and predicted effect on equity in the population. Assessment was made by experts' answering three questions (see Box 2 for a list of the questions) per criterion according to the conceptual framework of Rudan et al. [5,11]. This process yielded five intermediate scores, all ranging between 0% and 100%. The exact methods of computing intermediate scores are explained elsewhere [5,11].

**Activities of the larger reference group.** To ensure that the assessment of the research priorities is combined with a view of the wider society, the relative weights for each criterion were measured from 30 stakeholders' representatives from the larger reference group (LRG). LRG members included academics from three Cape Town universities, members of the public, government representatives, clinical psychologists, and other professionals. In choosing LRGs, we used a convenience sample attempting to secure a diverse mix of researchers, clinicians, professionals, academics, and members of the public and allowed them to express their opinions through an interview and as a quantitative score.

LRG members were first told the elements of the process, then asked for feedback, and eventually asked to rank those five criteria from the most important within the South African context (rank 1) to the least important (rank 5). The criterion of equity received the highest average rank (2.31), followed by efficacy and effectiveness (2.75); deliverability, affordability, and sustainability (2.94); maximum potential for mortality burden reduction (3.28); and answerability (3.72). These observed average ranks were then turned into weights by dividing the expected average rank in the situation of equal importance of all five criteria (which is 3.00) by the observed average rank. This simple procedure gives weights for the intermediate scores. Weighted means of intermediate scores were then computed to derive the final “research priority score” (RPS) for each research option.

**Box 1. Process for Setting Research Priorities**

**1. GATHERING OF TECHNICAL EXPERTS AND DEFINITION OF CONTEXTS**

- Initiation of the process of priority setting (a governmental department, a nongovernmental organization, or a research institution) and gathering of a group of technical experts
- Creation of definitions for geographic scale (global or national), time period (e.g., 5 or 10 years), target population (child health in our case), and targeted disease burden (e.g., pneumonia, HIV)

**2. LISTING RESEARCH OPTIONS SYSTEMATICALLY BY DOMAIN OF HEALTH RESEARCH**

The three domains of health research are:

- Health policy and systems research options (to improve efficiency of health systems already in place)
- Research options to improve existing interventions (affordability and deliverability)
- Research options to develop entirely new health interventions

**3. SCORING OF ALL LISTED RESEARCH OPTIONS BY CRITERION**

Experts in the TWG score the listed research options against five criteria:

- Likelihood that question can be answered in an ethical manner
- Likelihood of efficacy and effectiveness
- Likelihood of deliverability and affordability
- Maximum potential for disease burden reduction
- Likely impact of equity in population

**4. ADDRESSING STAKEHOLDERS' VALUES**

The LRG defines weights, which are placed on the five scores. The final RPS (0%–100%) is computed as weighted mean of intermediate scores

**5. PROGRAMME BUDGETING AND MARGINAL ANALYSIS; ADVOCACY**

- For each research option, its “value” in terms of the five criteria is combined with its proposed cost (in US$); program budgeting and marginal analysis derives optimal mix of options to be funded
- Based on this selection, the TWG advocates making the priorities and rationales accessible to the public; implements mechanisms for decision review; advocates for the implementation of identified priorities; and evaluates and improves the process based on feedback

## Outcomes

The final results of the scoring process of the technical experts (top 20 and bottom 10) are shown in Table 1. In this table, the scored research options are ranked by their final RPS multiplied by 100, which gives a range of score values between 0 and 100. This score takes into account the scores from technical experts, based on five criteria relevant to priority setting, and the weights defined by the LRG. The ranks in parenthesis indicate RPSs before weighting by the LRG. The final RPSs for the 63 research options ranged from 88.6 to 45.6, with the lowest score of 30.8 being an outlier. This range shows substantial variation between the research options in their likelihood to address the five criteria, as assessed by the TWG and LRG, and indicates that the methodology has the power to discriminate among many competing research options using a simple conceptual framework with 15 questions.

**Table 1:**
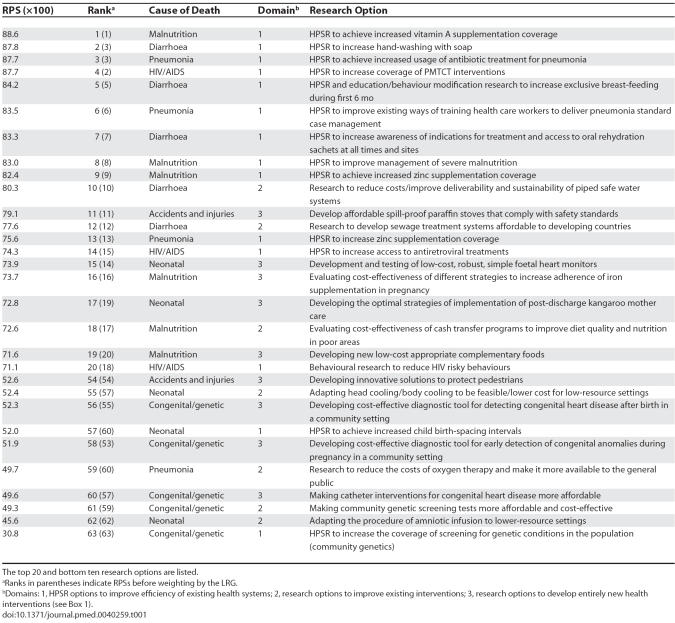
The Final Research Priority Scores and Ranks of 63 Research Options after Application of CHNRI Methodology to Address the Burden of Child Mortality in the Present South African Context

**Box 2. Questions That Technical Experts Apply to Listed Research Options**

**CRITERION 1: ANSWERABILITY AND ETHICS**

Q.1.1. Would you say the research question is well framed and end points are well defined?

Q.1.2. Based on (i) the level of existing research capacity in proposed research, and (ii) the size of the gap from current level of knowledge to the proposed endpoints, would you say that a study can be designed to answer the research question and to reach the proposed end points of the research?

Q.1.3. Is it likely that a study designed to answer the proposed research question would be granted ethical approval?

**CRITERION 2: EFFICACY AND EFFECTIVENESS**

Q.2.1. Based on the best existing evidence and knowledge, would the intervention that would be developed/improved through proposed research be efficacious?

Q.2.2. Based on the best existing evidence and knowledge, would the intervention that would be developed/improved through proposed research be effective?

Q.2.3. If the answers to either of the previous two questions are positive, would you say that the evidence upon which these opinions are based is of high quality?

**CRITERION 3: DELIVERABILITY, AFORDABILITY, AND SUSTAINABILITY**

Q.3.1. Taking into account the level of difficulty with intervention delivery from the perspective of the intervention itself (e.g., design, standardisability, safety), the infrastructure required (e.g., human resources, health facilities, communications, and transport infrastructure) and users of the intervention (e.g., need for change of attitudes or beliefs, supervision, existing demand), would you say that the end points of the research would be easily deliverable within the context of interest?

Q.3.2. Taking into account the resources available to implement the intervention, would you say that the end points of the research would be easily affordable within the context of interest?

Q.3.3. Taking into account government capacity and partnership requirements (e.g., adequacy of government regulation, monitoring, and enforcement; governmental intersectoral coordination; partnership with civil society and external donor agencies; favourable political climate to achieve high coverage), would you say that the end points of the research would be easily sustainable within the context of interest?

**CRITERION 4: MAXIMUM POTENTIAL FOR DISEASE BURDEN REDUCTION**

Q.4.1. Taking into account the results of conducted intervention trials (percentage reduction of disease burden in the intervention group in comparison to the control group) or for the new interventions, the proportion of avertable burden under an ideal scenario (computed from the knowledge of prevalence of risk factors targeted by future intervention and their relative risks, as “potential impact fraction”), would you say that the successful attainment of research end points would have a capacity to remove more than 10% of disease burden?

Q.4.2. More than 20% (or modify as appropriate per disease/condition)? Q.4.3. More than 40% (or modify as appropriate per disease/condition)?

**CRITERION 5: EQUITY IN ACHIEVED DISEASE BURDEN REDUCTION**

Q.5.1. Would you say that the present distribution of the disease burden affects mainly, or almost entirely, the underprivileged in the population?

Q.5.2. Would you say that mainly the underprivileged, or at least all segments of society equally, would be the most likely to benefit from the results of the proposed research after its implementation, rather than primarily the privileged?

Q.5.3. Would you say that the proposed research has the overall potential to improve equity in disease burden distribution in the long term (e.g., 10 years)?

Among the ten research options that received the highest RPSs (80.3 or greater), nine of them address “domain 1” of health research—HPSR for improving efficiency with the interventions that are already in place. Four among the top ten options addressed diarrhoea, three addressed malnutrition, two addressed pneumonia, and one addressed HIV/AIDS. The priority was generally given to the research on more efficient delivery of already existing, cost-effective interventions: vitamin A supplementation, hand washing, antibiotics for pneumonia, PMTCT, and breast-feeding.

Among the addressed diseases and conditions, the most represented in the top were those that contribute most to the child mortality in South Africa. An exception to this rule was a relative under-representation of research options, with the exception of increasing PMTCT coverage, addressing the management of HIV/AIDS. Research options addressing neonatal causes of death were also under-represented relative to their share in child mortality burden in South Africa.

This group of priorities is followed by research on improving interventions that could become highly efficient if they could be made more affordable, deliverable, and sustainable, such as antiretroviral treatment for HIV/AIDS, or piped safe water systems and sewage treatment systems to help prevent diarrhoea. Another large group of priorities includes those addressing diseases and conditions with a large effect on mortality, but for which there are no existing interventions that could achieve high population coverage in an equitable way. Examples of such priorities are the development and testing of low-cost, robust, simple foetal heart monitors and developing the optimal strategies of implementation of post-discharge “kangaroo mother care” (skin-to-skin contact between mother and infant). In such cases, an attempt to develop entirely new interventions was given greater priority than research on creative scaling up or improving the existing interventions.

Among the 21 research options from the bottom third of the final list of rankings that involved all 63 research options, seven addressed congenital and genetic causes of child deaths, while another four addressed accidents and injuries. This low prioritization is not surprising, given their relatively low burden of disease and lack of cost-effective interventions. However, eight research options addressed pneumonia and neonatal conditions in this section, both of which cause significant mortality. The concerns over the proposed research to develop new interventions to address pneumonia were answerability, affordability, and impact on equity. The research options of very low RPS addressing neonatal causes of deaths were focused on more efficient delivery and improvement of existing interventions. Those scores reflect very low confidence of the technical experts in the potential value of health interventions that already exist to address neonatal causes of death.

## Discussion

The results of this prioritization exercise suggest that child health research funding in South Africa should concentrate on HPSR options, especially those related to diarrhoea, pneumonia, and malnutrition. Our results are in line with the findings from Anthony Costello and colleagues' recent priority-setting exercise with international experts that used the Delphi method [12]. The results probably reflect the fact that, although pneumonia and diarrhoeal disease represent 38% of the global burden of disease in children, only an estimated 0.2% of the total funding spent on research and development is allocated to these conditions [13]. Furthermore, a recent investigation found that 97% of research grants from the largest not-for-profit sources of funds for health research (the US National Institutes of Health and the Bill and Melinda Gates Foundation) were for developing new technologies, which could reduce child mortality by 22%, a reduction one-third of what could be achieved if existing technologies were fully realized [14].

Research for HIV/AIDS and neonatal conditions did not feature as highly despite being the areas of a significant burden of disease. For HIV/AIDS this lower prioritization was due to the relative lack of cost-effective interventions to fight paediatric AIDS that could realistically achieve high population coverage, such as a long-awaited vaccine. However, a research option “development of HIV vaccine” was ranked 37th by technical experts and 39th after adjustments of the scores according to the values of stakeholders, because its answerability and effect on equity upon its initial implementation achieved very low scores in comparison to already existing cost-effective interventions available for other diseases. Neonatal conditions also were given lower value due to the lack of existing cost-effective interventions. This lack also explains why the most highly ranked research options addressing neonatal causes of death, at ranks 15 and 17, were those addressing the development of entirely new interventions, in this case “development and testing of low-cost, robust, simple foetal heart monitors” and “developing the optimal strategies of implementation of post-discharge kangaroo mother care”, respectively.

A recent systematic review of national health priorities for child health research in sub-Saharan Africa concluded that there are few systematically developed national research priorities [15]. The review also found that in the rare cases in which a country does have such priorities, children's interests may be distorted by processes that combine all age groups [15]. There are no data of which we are aware that detail how research funding on child health is spent in South Africa. Our discussions with child health researchers suggest that the vast majority of funding is directed toward research addressing HIV/AIDS, and with substantial amounts being directed toward HIV vaccine research.

## Limitations of Our Approach

We do not believe the predominance of HPSR is due to a bias of methodology. In the context of the high remaining burden of child mortality in South Africa [8] and the presence of cost-effective interventions and sufficient available resources to implement them, we expected that the methodology should highlight the issues of improved delivery and increased coverage of those interventions as an immediate priority. Furthermore, among the 16 research options at the bottom of the list of rankings, four of them (25%) are HPSR options (including the outlier at the bottom). The results may still be biased by the choice of the technical experts and LRG. However, even if there were such a bias, the methodology transparently presents the input from every expert on every criterion, so the input would be reviewed and challenged by other experts, and the feedback loop within the methodology would take those changes into account. This process of review also helps to identify the points of widespread agreement and the points of controversy, thereby directing and focusing the discussion on research prioritization on the key issues.

The selection of research topics was restricted to the top seven causes of death in children under age 5 in South Africa, which were jointly responsible for more than 90% of annual deaths in this age group [8]. Furthermore, for each selected cause of death, an equal number of research options addressing three domains of health research was proposed for scoring, to avoid favouring any of the domains. This structure led to a total of 63 research options to score (3 options × 3 domains × 7 causes), which was feasible because all technical experts managed to complete the scoring process within 1–3 days. The time they initially invested into selecting those options ensured that the final list with 63 options did not try to present all theoretical research possibilities, rather the options that they felt were most likely to be identified as priorities when evaluated against all the other options using the same structured process and a set of criteria. Finally, even though the methodology takes into account the general values of an LRG it would be useful to have additional criteria that take into account existing government priorities [16].

The challenge of this exercise was then to avert child mortality burden as quickly as possible through better investments in health research. Therefore, our exercise does not consider prioritization of investments in health care, which is perhaps even more important, but also an order of magnitude more complex. We acknowledge that there is also an important avenue of research that lies on the border between health research and health care policy development and planning that should be considered. We clearly need research to identify coherent approaches to strengthen health systems. Operational research does compete with other national and global research priorities in terms of funding and, perhaps more importantly, in terms of technical expertise. Neither local communities nor subnational health departments in South Africa presently have the capacity to perform many of these research activities, and, unless more national and global support is provided, local operational research will continue to be ignored. Defining research priorities globally within the HPSR domain needs a careful and well-planned effort coordinated by leading experts, and we felt it was beyond the scope of this particular study, which piloted the methodology at the national level.

## Conclusion

The main advantages of the CHNRI methodology presented in this article over the alternative approaches can be summarized as: (1) being systematic in listing and scoring competing research options, thus limiting the influence of personal biases on the outcome; (2) being transparent in regard to the input and contribution of everyone involved in deciding the research priorities; (3) preventing one or a few individuals from dominating the process; (4) presenting a simple quantitative outcome (RPS); (5) simultaneously evaluating different domains of health research using the same set of criteria; and (6) incorporating the opinion of stakeholders and wider public. The methodology proved to be a feasible and transparent approach toward setting priorities in child health research investments in South Africa.

Still, the methodology is new and further possibilities of improvement are certainly possible. Some of them include: (1) improving strategies to ensure better representativeness of the experts who undertake the scoring process and stakeholders who introduce weights to intermediate scores; (2) evaluating the outcome of the methodology by comparing it to the results of other methods; (3) validating the results of different priority-setting methods by showing that they indeed affect disease burden and reduce it in an equitable way; (4) presenting the outcomes of the process by publication, by inviting comments from the wider research community, and by using the feedback loop to modify the outcomes based on justified comments.

Our intention is to now use the derived scores for the final steps shown in Box 1 to perform program budgeting and marginal analysis at the national level. Marginal analysis allocates available funding resources to identified priorities to maximize the benefits. This process results in maximally equitable reduction of disease burden by choosing the optimal “mix” of funded research options within the budgetary constraint. We also intend to make the results accessible to the wider public, to implement mechanisms for reviewing the scores and decisions, to advocate and implement the identified priorities, and to evaluate and improve this process based on feedback.

## Abbreviations

CHNRI: Child Health and Nutrition Research Initiative
HPSR: health policy and systems research
LRG: larger reference group
PMTCT: prevention of mother-to-child HIV transmission
RPS: research priority score
TWG: technical working group
